# Dietary Modulation Alters Susceptibility to Listeria monocytogenes and Salmonella Typhimurium with or without a Gut Microbiota

**DOI:** 10.1128/mSystems.00717-21

**Published:** 2021-11-02

**Authors:** Mathis Wolter, Alex Steimle, Amy Parrish, Jacques Zimmer, Mahesh S. Desai

**Affiliations:** a Department of Infection and Immunity, Luxembourg Institute of Health, Esch-sur-Alzette, Luxembourg; b Faculty of Science, Technology and Medicine, University of Luxembourg, Esch-sur-Alzette, Luxembourg; c Odense Research Center for Anaphylaxis, Department of Dermatology and Allergy Center, Odense University Hospital, University of Southern Denmark, Odense, Denmark; Duke University School of Medicine

**Keywords:** colonic mucus layer, dietary fiber, gut microbiota, *Listeria monocytogenes*, pathogen susceptibility, *Salmonella* Typhimurium

## Abstract

Food safety has considerably improved worldwide, yet infections with foodborne human enteric pathogens, such as *Listeria* spp. and Salmonella spp., still cause numerous hospitalizations and fatalities. Since dietary alterations, including fiber deficiency, might impact the colonization resistance mediated by the gut microbiome, studying the diet–microbiome–pathogen axis holds promise in further understanding the pathogenesis mechanisms. Using a gnotobiotic mouse model containing a 14-member synthetic human gut microbiota (14SM), we have previously shown that dietary fiber deprivation promotes proliferation of mucin-degrading bacteria, leading to a microbiome-mediated erosion of the colonic mucus barrier, which results in an increased susceptibility toward the rodent enteric pathogen Citrobacter rodentium. Here, we sought to understand how a low-fiber diet affects susceptibility to Listeria monocytogenes and Salmonella enterica serovar Typhimurium by using our 14SM gnotobiotic mouse model in BALB/c and C57BL/6 mouse backgrounds, respectively. Intriguingly, and in contrast to our results with C. rodentium, we observed that depriving mice of dietary fiber protected them from infections with both pathogens, compared to mice fed a standard chow. The microbiome delayed the overall pathogenicity compared to the onset of disease observed in germfree control mice. Nevertheless, we observed the same effect of diet on germfree mice, suggesting that the susceptibility is directly driven by the diet itself even in the absence of the gut microbiome. Our study points out an important observation, namely, that dietary fiber plays a crucial role in either the host’s susceptibility, the virulence of these pathogens, or both. It would be judicious to design and interpret future studies on this basis.

**IMPORTANCE** The human enteric pathogens Listeria monocytogenes and Salmonella Typhimurium are employed as classical models in rodent hosts to understand the pathogenesis mechanisms of foodborne pathogens. Research in the past decade has stressed the importance of the gut microbial composition in modulating susceptibility to these pathogens. The results of our study—using gnotobiotic mice and germfree control animals—additionally suggest that the dietary fiber components can dominate the impact of enteropathogenic virulence over the pathogenicity-modulating properties of the gut microbiome. The significance of our research is that there is a need to carefully choose a certain chow when performing the enteropathogen-associated mouse experiments and to cautiously match the rodent diets when trying to replicate experiments across different laboratories. Finally, our data underscore the importance of using germfree control animals to study these pathogens, as our findings would have been prone to misinterpretation in the absence of these controls.

## OBSERVATION

The gut microbiome confers colonization resistance against invading pathogens by nutrient competition and by maintaining the host’s immune homeostasis and the mucosal barrier’s integrity (1). However, a deficiency of dietary fiber might negatively affect these host-beneficial properties of the microbiome (2). Since dietary fiber consumption in Western countries is below the recommended intake of 25 to 35 g per day (3), such dietary habits might contribute to the observed incidence of enteric pathogen infections in the Western world. Using a well-characterized 14-member synthetic human gut microbiota (14SM) in gnotobiotic mice, we previously demonstrated that dietary fiber deprivation leads to an increase in the mucin-degrading activity of the gut microbiome, which excessively erodes the colonic mucus barrier (4). We further showed that the reduced mucus barrier enhances susceptibility to infection with Citrobacter rodentium (4), a rodent pathogen used to model human enteropathogenic and enterohemorrhagic Escherichia coli infections (5). Since the intestinal mucus barrier is a first line of innate defense (1), here, we hypothesized that diet-induced mucus erosion might also increase susceptibility to other enteric pathogens.

Food safety has increased considerably in recent years, yet foodborne enteric pathogens, such as *Listeria* spp. and Salmonella spp., remain a major source of disease, even in industrialized countries (6, 7). Since dietary alterations, including fiber deficiency, might alter colonization resistance by the gut microbiome to enteric pathogens (1), understanding the interconnections in the diet–microbiome–pathogen axis might help to shed light on hitherto unexplored pathogenesis mechanisms. It has previously been shown that mice lacking the *Muc2* gene, which encodes the major constituent glycoprotein of the colonic mucus layer, are more susceptible to Listeria monocytogenes and Salmonella enterica serovar Typhimurium infections (8, 9). Notably, a similar increase in susceptibility was observed for C. rodentium in *Muc2^−/−^* mice (10), a result that we could recapitulate in wild-type, fiber-deprived mice with a reduced mucus barrier (4). Thus, we leveraged our 14SM gnotobiotic model to investigate how dietary fiber deprivation and/or an eroded mucus barrier affects the host’s susceptibility toward infections with the intracellular enteric pathogens L. monocytogenes and *S.* Typhimurium.

**FIG 1:**
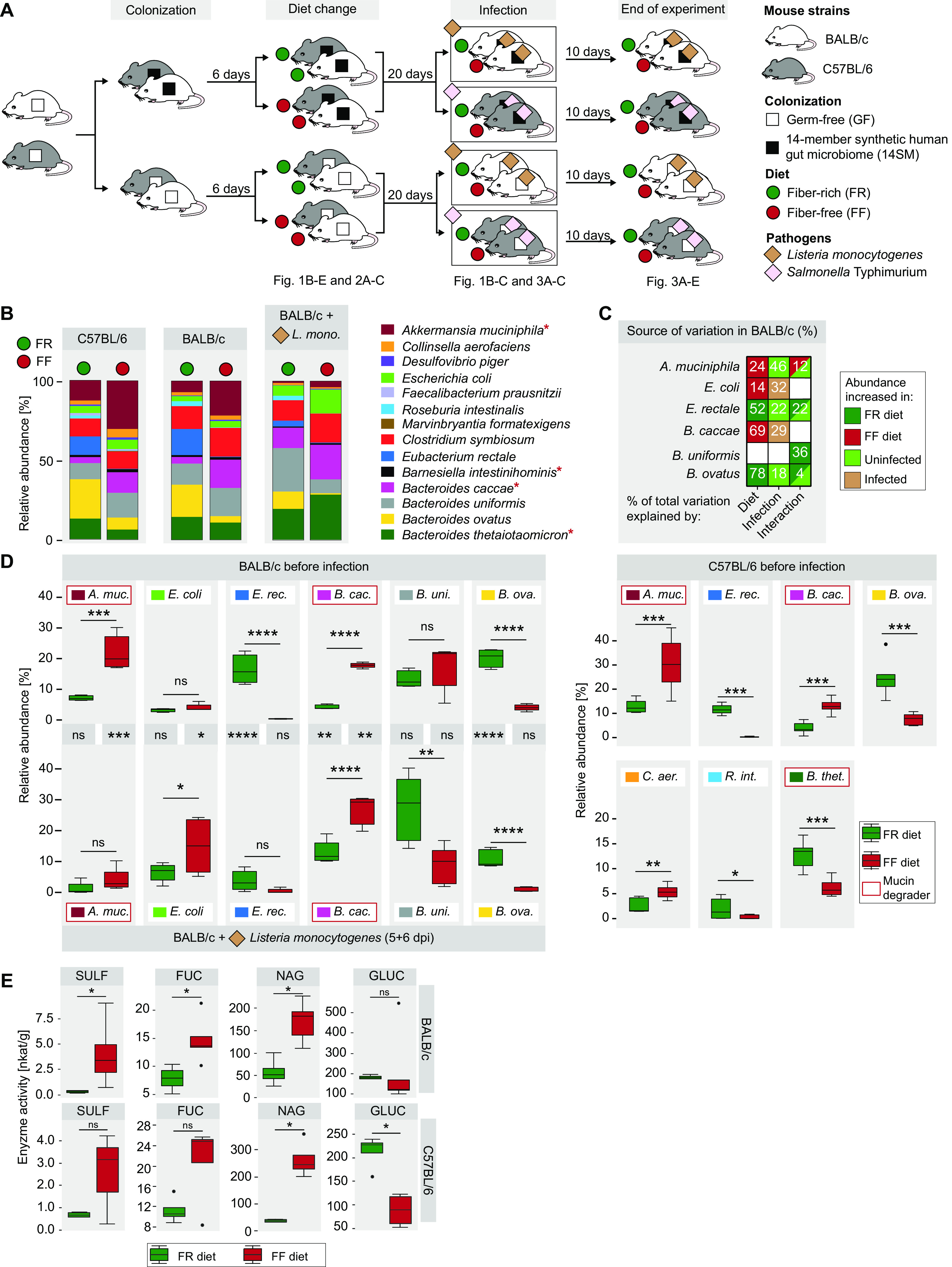
Fiber deprivation increases the abundance and activity of mucin-degrading gut bacteria in both BALB/c and C57BL/6 mice. (A) Experimental timeline. Half of the 6- to 10-week-old, age-matched, GF BALB/c and C57BL/6 mice were gavaged with the 14-member gut microbiota (14SM) on two consecutive days, while the other half were maintained GF. Six days after the gavage, half of the mice from the GF and 14SM groups continued on the FR diet, while the other half were switched to the FF diet. The mice were maintained on their respective diets for 20 days, and then the BALB/c mice were infected with Listeria monocytogenes and the C57BL/6 mice were infected with Salmonella Typhimurium, after which the mice were observed for another 10 days. (B) Relative bacterial abundances before infection and for BALB/c mice after infection, determined by qPCR on DNA extracted from fecal pellets. While some low-abundance bacteria might not be visible in the figure, all 14 bacteria were detected. Red asterisks denote known mucin-degrading bacteria. (C) Sources of variation of the bacterial relative abundances in BALB/c mice, with consideration of the two variables, diet and infection status, determined by two-way ANOVA. The percentage of total variation due to diet, infection with L. monocytogenes, or the interaction of these two factors is represented in each cell. The trend of the shift in the abundance by a given factor is represented by the color of each cell. (D) Members of the 14SM whose abundance significantly changed due to diet, determined in BALB/c mice before and after the infection with L. monocytogenes and in C57BL/6 before infection with *S.* Typhimurium. Significance labels between the top and bottom panels represent comparisons between a given group before and after infection. For the Tukey box plot, BALB/c mice were analyzed by two-way ANOVA with a Tukey-Kramer *post hoc* test and C57BL/6 mice were analyzed by a Mann-Whitney test. (E) Glycan-degrading enzyme activities of the gut microbiome in stool samples determined by *p*-nitrophenyl glycoside-based enzyme assays. Sulfatase (SULF), α-fucosidase (FUC), and β-*N*-acetyl-glucosaminidase (NAG) are key mucin-degrading enzymes, while β-glucosidase (GLUC) serves as a control for general glycan-degrading activity. Tukey box plot values were determined by the Wilcoxon rank sum test. There were 5 BALB/c mice/group. For the C57BL/6 mice, there were 7 GF FR mice per group; for the other groups, there were 8 mice per group. ns, nonsignificant; *, *P* < 0.05; **, *P* < 0.01; ***, *P* < 0.001; ****, *P* < 0.0001. *L. mono.*, L. monocytogenes; *A. muc.*, A. muciniphila; *E. rec.*, E. rectale; *B. cac*., B. caccae; *B. uni.*, Bacteroides uniformis; *B. ova.*; Bacteroides ovatus; *C. aer.*, Collinsella aerofaciens; *R. int.*, Roseburia intestinalis; *B. thet.*, Bacteroides thetaiotaomicron.

For this purpose, we employed BALB/c and C57BL/6 mice for infections with L. monocytogenes and *S.* Typhimurium, respectively; the choice of the host strains for each pathogen is based on previous studies (12–14). We colonized 6- to 10-week-old, germfree (GF) mice with the 14SM community and confirmed colonization of all 14 strains by quantitative PCR (qPCR) using strain-specific primers as described previously (4, 15). For 6 days after colonization, the mice were kept on a standard mouse chow, which we call a fiber-rich (FR) diet. After 6 days, half of the mice were switched to a fiber-free (FF), high-sugar diet. After an additional 20 days, BALB/c mice were infected via intragastric gavage with 10^9^ CFU of L. monocytogenes and C57BL/6 mice were infected with 10^8^ CFU of *S.* Typhimurium. The infection progress was monitored for up to 10 days (Fig. 1A). As a control for the 14SM-colonized mice, age- and sex-matched GF BALB/c and C57BL/6 mice were used. These GF controls were also fed either the FR or the FF diet before being subjected to L. monocytogenes and *S.* Typhimurium infection (Fig. 1A).

Throughout the feeding period before the pathogen infection, neither FR diet- nor FF diet-fed mice exhibited any obvious physiological abnormalities, irrespective of whether they were 14SM colonized or not. Fiber deprivation significantly altered the gut microbiota compositions of BALB/c and C57BL/6 mice (Fig. 1B). Infection of C57BL/6 mice caused severe diarrhea, making the analysis of their fecal matter impossible. Thus, analysis of the gut microbiota of infected mice could be performed only for BALB/c mice, which showed that the infection majorly influenced the microbiota composition (Fig. 1B). All bacterial members within the 14SM that shifted in response to the diet switch were further affected by the infection with the pathogen (Fig. 1C). In line with our previously published study with 14SM-colonized Swiss Webster mice (4), fiber deprivation increased the relative abundances of mucin-degrading bacteria, such as Akkermansia muciniphila and Bacteroides caccae (4), whereas the abundances of the fiber-degrading strains, such as Bacteroides ovatus and Eubacterium rectale (4), significantly decreased (Fig. 1D). Interestingly, the significantly increased abundance of A. muciniphila owing to the FF diet is nullified by its reduced abundance following the infection (Fig. 1C and D).

Unlike with C. rodentium, the primary infection site of the intracellular pathogens L. monocytogenes and *S.* Typhimurium is the small intestine and not the colon (12, 16). Nevertheless, a recent study highlighted the role of the colon as the main site for establishing the systemic spread of L. monocytogenes in *Muc2^−/−^* mice (8). Accordingly, the expansion of mucin-degrading commensals in FF diet-fed mice (Fig. 1D) prompted us to investigate the activities of certain bacterial mucin glycan-degrading enzymes, which can be used as a proxy for the microbiome-mediated erosion of the mucus barrier (17). Using fecal samples, we detected increased activities of key mucin glycan-degrading bacterial enzymes (18), such as sulfatase (SULF), α-fucosidase (FUC), and β-*N*-acetylglucosaminidase (NAG), in FF diet-fed BALB/c and C57BL/6 mice compared to those in FR diet-fed mice (Fig. 1E), although in BALB/c mice, the trends were not significant for SULF and FUC. On the other hand, the activity of β-glucosidase (GLU)—an enzyme that indicates microbial plant fiber metabolism (19)—significantly decreased in FF diet-fed C57BL/6 mice (Fig. 1E). Overall, while activities were less pronounced in C57BL/6 mice, our results suggest that fiber deprivation leads to increased activities of enzymes essential for mucin glycan degradation (Fig. 1E). We have previously shown using Swiss Webster mice that the increased mucin-degrading bacteria and the microbial enzymes that target the colonic mucins result in a reduced mucus barrier (4); therefore, here, we suspect a similar mucus barrier reduction in the C57BL/6 and BALB/c mice.

**FIG 2:**
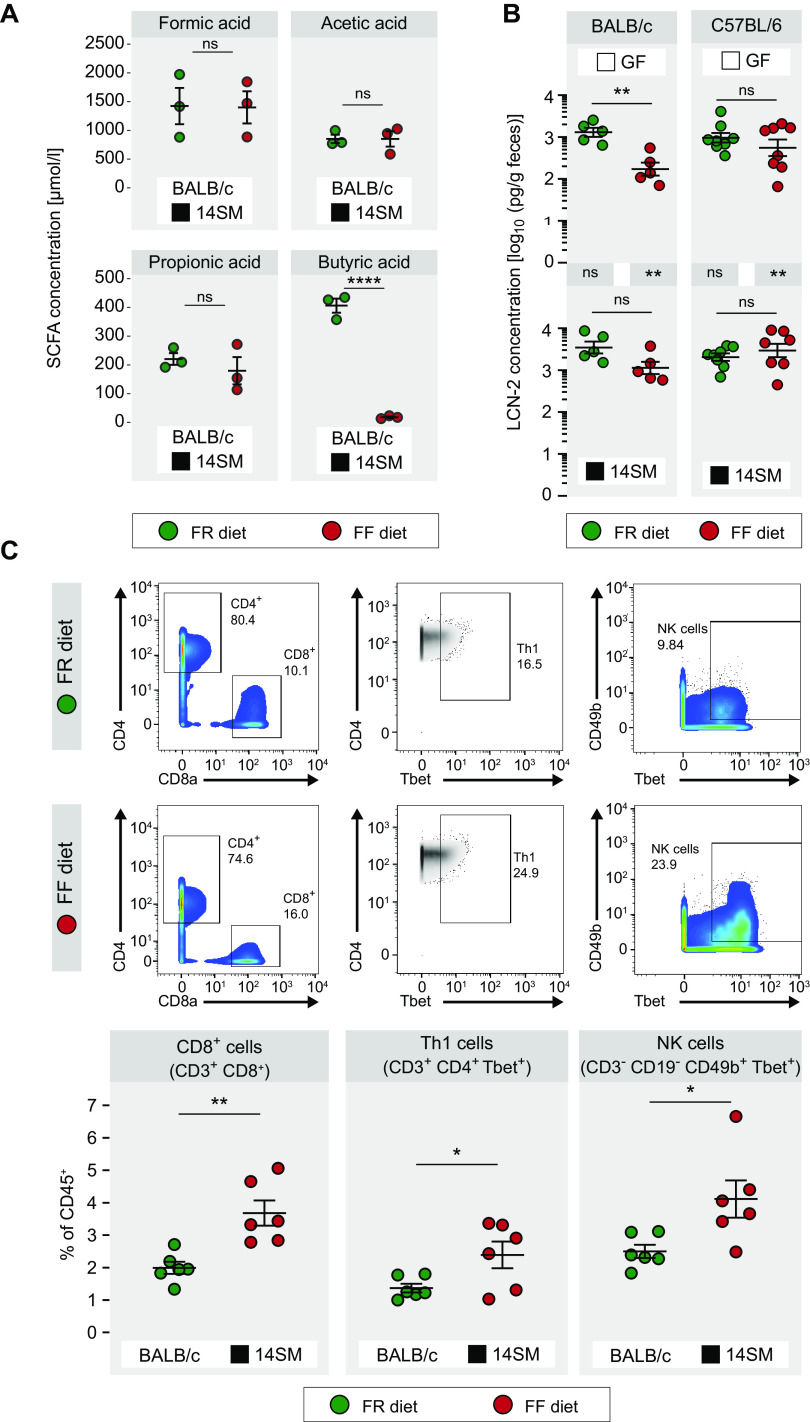
Fiber deprivation can prime the host against infection. (A) Short-chain fatty acid concentrations in cecal contents of uninfected BALB/c mice. Error bars represent standard errors of the means (SEM) from an unpaired, two-tailed *t* test (*n* = 3 mice/group). (B) Fecal LCN-2 levels determined by ELISA on the day before the pathogen infection. Significance labels between the top and bottom panels represent comparisons between GF and 14SM mice of a given dietary group. Error bars represent SEM from a two-way ANOVA with a Tukey-Kramer *post hoc* test. There were 5 BALB/c mice/group. For C57BL/6 mice, there were 7 GF FR diet-fed mice per group; in other groups, there were 8 mice per group. (C) CD8^+^, Th1, and NK immune cell populations in colonic lamina propria of uninfected BALB/c mice determined using time of flight mass cytometry (CyTOF). Gating plots (top) show percentages of cells of the previously gated population (the percentage of CD3^+^ cells for CD8^+^ cells, the percentage of CD4^+^ cells for Th1 cells, and the percentage of CD19^–^ cells for NK cells). In contrast, dot plots (bottom) are represented as percentages of all CD45^+^ cells. Error bars represent SEM from an unpaired, two-tailed *t* test (*n* = 6/mice group, two independent experiments). ns, nonsignificant; *, *P* < 0.05; **, *P* < 0.01; ***, *P* < 0.001; ****, *P* < 0.0001.

We then analyzed the short-chain fatty acid (SCFA) concentrations, as they serve as important regulators of mucosal barrier integrity (20). Nevertheless, despite the significant changes in microbial composition and activity, butyrate was the only SCFA with a significantly altered concentration (Fig. 2A). As low butyrate levels indicate an impaired mucosal barrier (20) and considering the suggested diet-induced impairment of the colonic mucus layer (Fig. 1E), we determined potential diet-induced colonic inflammation via detection of fecal lipocalin-2 (LCN-2) levels, which is considered a biomarker for low-grade inflammation (21). Significantly increased levels of LCN-2 were observed only in FR diet-fed GF BALB/c mice, not in their FF diet-fed counterparts (Fig. 2B). In contrast, in our previous study, 14SM-colonized Swiss Webster mice showed increased LCN-2 levels when fed the FF diet (4), suggesting that dietary fiber-mediated colonic baseline inflammation is likely dependent on the rodent genetic background. Furthermore, we detected higher concentrations of LCN-2 in 14SM-colonized and FF diet-fed mice than in their GF counterparts (Fig. 2B). However, when we assessed the local immune phenotype by time-of-flight mass cytometry (CyTOF)-based profiling of CD8^+^, Th1, and NK immune cell populations in the colonic lamina propria of uninfected 14SM-colonized BALB/c mice, all of these immune cells were increased in the FF diet-fed mice (Fig. 2C). These data indicate an overall increased type 1 inflammatory immune response under FF diet conditions, which seems to be regulated independently of the LCN-2 pathways.

**FIG 3:**
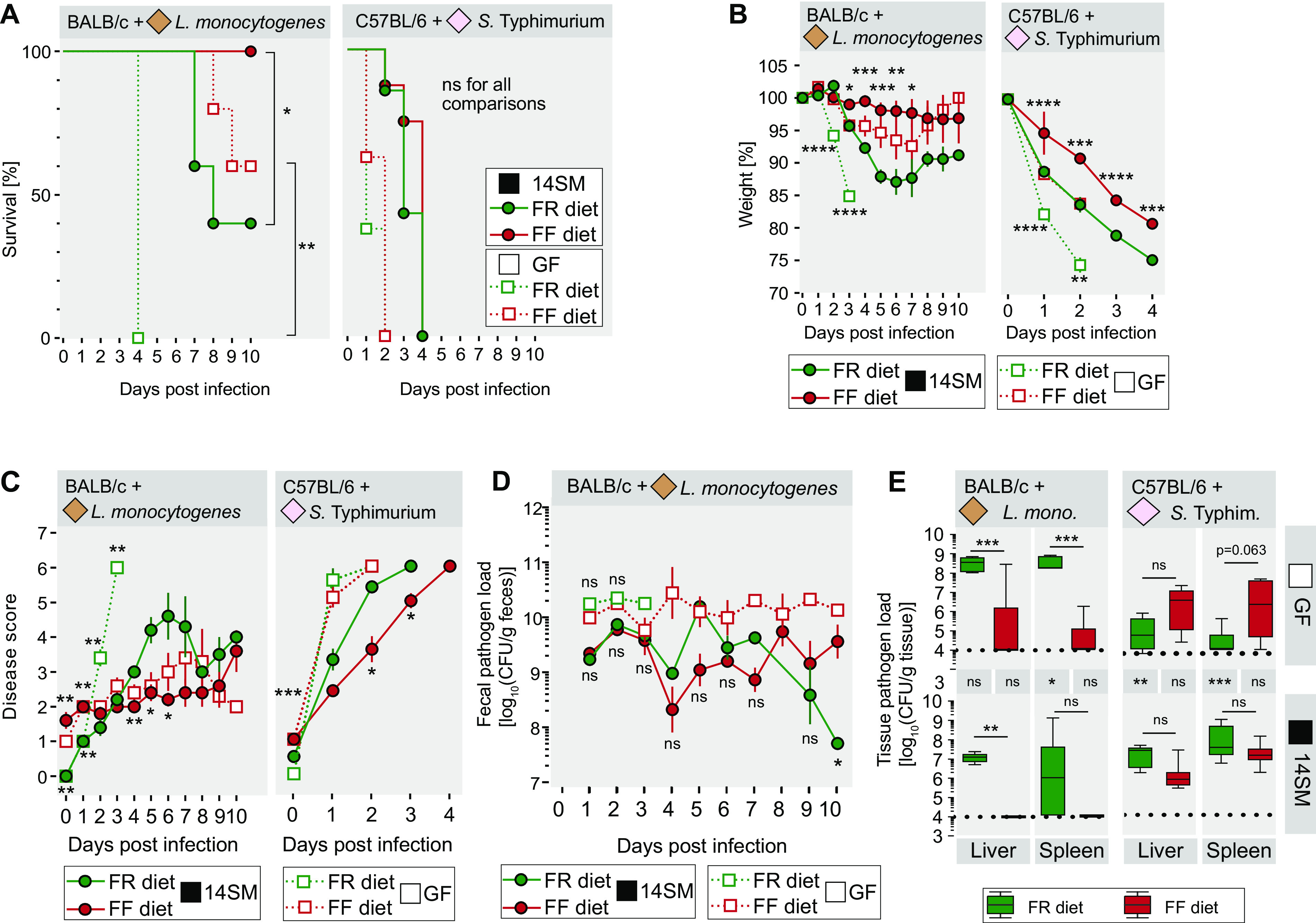
Fiber deprivation protects against L. monocytogenes and *S.* Typhimurium with or without the presence of a gut microbiota. (A) Survival curve of enteropathogen-infected mice determined by a log rank test between both diets of the GF or 14SM group. (B) Weight change of the enteropathogen-infected mice. The day 0 value was determined immediately before the gavage. Error bars represent SEM as determined by an unpaired, two-tailed *t* test between both diets of the GF (bottom significance labels) or 14SM (top significance labels) group; comparisons are not significant when the significance is not displayed. (C) Average disease scores attributed to each enteropathogen-infected group. The day 0 value was determined immediately before the gavage. Error bars represent SEM as determined by a Mann-Whitney test between both diets of the GF (top significance labels) or 14SM (bottom significance labels) group; comparisons are not significant when the significance is not displayed. (D) Fecal L. monocytogenes loads of BALB/c mice during the 10 days of infection. Depending on the sampling day, 1 to 5 samples per group were obtained and evaluated. The fecal *S.* Typhimurium load in C57BL/6 mice could not be determined, as the mice did not consistently provide fecal material due to the severe disease. Tukey box plot values are from an unpaired, two-tailed *t* test. (E) Pathogen loads of liver and spleen tissues on the day that each mouse was euthanized. Samples below the measurable threshold of 10^4^ CFU (dotted black line) were considered 10^4^. Significance labels between the top and bottom panels represent comparisons between GF and 14SM mice of a given dietary group. Tukey box plot values were determined by two-way ANOVA with a Tukey-Kramer *post hoc* test. There were 5 BALB/c mice per group. For the C57BL/6 group, there were 7 GF FR diet-fed mice per group; for other groups, there were 8 mice per group. Green, FR diet-fed mice; red, FF diet-fed mice; smooth lines, 14SM mice; dotted lines, GF mice. ns, nonsignificant; *, *P* < 0.05; **, *P* < 0.01; ***, *P* < 0.001; ****, *P* < 0.0001.

After a 20-day feeding period, we infected both mouse strains with their respective pathogens (Fig. 1A). Body weight and disease scores of all mouse groups were assessed daily for up to 10 days postinfection (dpi). The lethality of L. monocytogenes-infected GF FR diet-fed BALB/c mice reached 100% by 4 dpi, while their FF diet-fed counterpart provided a significantly higher survival rate (Fig. 3A). Similarly, 14SM-colonized FF diet-fed BALB/c mice had a significantly higher survival rate than their FR diet-fed counterparts, and intriguingly, all 14SM-colonized FF diet-fed BALB/c mice survived the infection (Fig. 3A). In accordance with previous reports stating that mice harboring an intestinal microbiota are less susceptible to L. monocytogenes infections than GF mice (11), 14SM-colonized BALB/c mice generally provided increased survival compared to that of the GF controls fed the same diet (Fig. 3A). In line with the course of the survival curves, weight loss in FR diet-fed and L. monocytogenes-infected BALB/c mice, either 14SM colonized or GF, was significantly higher than in the corresponding FF diet-fed groups (Fig. 3B). Daily assessed disease scores for all four L. monocytogenes-infected BALB/c mouse groups (Fig. 3C; see Table 1 for the disease-scoring scheme) underscore that susceptibility to L. monocytogenes infection is more dependent on the fiber content of the diet itself than on the microbiome. Interestingly, fecal L. monocytogenes loads did not significantly differ between both diets of the GF and 14SM groups, except at the final time point in the 14SM group (Fig. 3D), hinting at a faster clearance in 14SM FR diet-fed mice. Systemic dissemination of L. monocytogenes in BALB/c mice was assessed by the detection of CFU in liver and spleen (Fig. 3E). Unlike with the fecal pathogen levels, both FR diet-fed groups showed significantly increased dissemination of L. monocytogenes into the liver compared to that of their FF diet-fed counterparts (Fig. 3E). Similarly, dissemination into the spleen was significantly higher in GF FR diet-fed mice than in the FF diet-fed controls (Fig. 3E). These results suggest that feeding mice a fiber-free diet does not affect the growth of L. monocytogenes but hinders its translocation across the intestinal epithelium.

**TABLE 1:** Scoring system used to determine the disease severity of mice^*a*^

Category	Score
Body wt	
5–10% wt loss	1
11–15% wt loss	2
16–20% wt loss	3
≥20% wt loss	HEP
Pinched skin/dehydration	4

Coat condition	
Coat slightly unkempt	1
Slight piloerection	2
Marked piloerection	4

Body function	
Tachypnea	3
Dyspnea	5

Environment	
Loose stools or diarrhea	1
Blood in diarrhea	HEP

Behaviors	
Tense and nervous on handling	3
Markedly distressed on handling, e.g., shaking, vocalizing, aggressive	4

Locomotion	
Slightly abnormal gait/posture	1
Markedly abnormal gait/posture	4
Significant mobility problems or reluctance to move	HEP

Procedure-specific indicator	
Conjunctivitis	4

Implementation of HEP	
Total score	≥6

a Mice reaching the humane endpoint (HEP) were scored the maximum score of 6.

In contrast to L. monocytogenes-infected BALB/c mice, all *S.* Typhimurium-infected C57BL/6 mice died within 4 days of infection (Fig. 3A). There were no significant differences in survival rates between FR diet-fed and FF diet-fed GF mice or between 14SM-colonized mice fed the two different diets (Fig. 3A). Despite no significant differences in survival between *S.* Typhimurium-infected C57BL/6 mice fed different diets, weight loss in FR diet-fed 14SM-colonized mice, as well as in FR diet-fed GF C57BL/6 mice, was significantly increased compared to that of their FF diet-fed 14SM-colonized or GF mice (Fig. 3B). Disease scores of all mice reached the maximum possible score of 6 (Fig. 3C). Notably, GF mice were more susceptible to *S.* Typhimurium infection than 14SM-colonized mice, and FR diet-fed 14SM mice provided significantly higher disease scores than FF diet-fed 14SM-colonized mice. No significant differences in levels of dissemination of *S.* Typhimurium into the liver or spleen were detected (Fig. 3E). Interestingly, dissemination into the spleen and liver was significantly higher in 14SM-colonized mice than in GF mice (Fig. 3E).

Our data suggest that fiber deprivation has a protective effect against L. monocytogenes infections and to a reduced extent against *S.* Typhimurium. In contrast to Swiss Webster mice infected with cecum- and colon-targeting C. rodentium (4) and to *Muc2^−/−^* mice (8, 9), elevated microbial mucin degradation in BALB/c and C57BL/6 mice, as a consequence of fiber deprivation, did not promote susceptibility to the two enteropathogens. One potential explanation is that even a microbially eroded colonic mucus layer still provides sufficient protection to prevent the colon from becoming the locus for the systemic spread of these pathogens; alternatively, the direct effects of diet might simply dominate the impact of an eroded mucus layer. Overall, we determined a direct impact of dietary fiber components on host susceptibility to enteropathogenic infections, which seems to be rooted in a heightened translocation efficiency. Considering the potential cytotoxic environment resulting from the increased type 1 inflammatory immune response in the uninfected, FF diet-fed BALB/c mice compared to that of the FR diet-fed BALB/c mice (Fig. 2C), we hypothesize that this priming of the immune system provides protection against subsequent infection. Specifically, CD8^+^ T cells are known to play a crucial role in clearing L. monocytogenes infections (22, 23), and their increased percentage in the FF diet-fed 14SM-colonized mice (Fig. 2C) might at least partially explain why this group of mice is better protected from the infection.

Apparently, the 14SM could not protect the mice via colonization resistance, although the microbiome delayed the overall disease course and the pathogen load. Furthermore, we cannot exclude the possibility that the fiber types present in our FR diets promote pathogen virulence. Based on the ingredients provided by the supplier for this chow, β-glucans and arabinoxylans appear to be the dominant fibers. Indeed, a study in guinea pigs showed that supplementation with the dietary fibers pectin and inulin significantly increased the translocation of L. monocytogenes into the liver and spleen (24). However, this study also shows that supplementation with galacto-oligosaccharides and xylo-oligosaccharides decreased the translocation (24), indicating a fiber source-specific virulence modulation of L. monocytogenes. In this context, increased fiber consumption has previously been linked to both increased and decreased susceptibility (13, 25, 26) to *S.* Typhimurium infections, indicating that not only the presence or absence of dietary fiber in a mouse chow determines enteropathogen susceptibility but also the source or type of fiber is an essential factor. Despite many advantages of gnotobiotic mouse studies (27), the potential absence of interactions between specific commensal bacteria and pathogens such as *Prevotella* spp. with L. monocytogenes (14) or Mucispirillum schaedleri with *S.* Typhimurium (28) must be considered a limitation of our 14SM model. Moreover, gnotobiotic models might fail to provide a real-life picture of colonization resistance provided by a complex microbiome against both L. monocytogenes and *S.* Typhimurium infections (11, 12). Another potential caveat in comparing results obtained from our FR and FF diets might be the higher amount of glucose in the FF diet, especially given that the main infection site of these pathogens is the small intestine (4). It is difficult to disentangle the effect of low fiber and high glucose; although we showed that the fecal load of L. monocytogenes was unaffected by the diet, we cannot fully exclude the potential effect of sugar on the infectiousness of the pathogens.

The intriguing, direct impact of dietary fiber on the increased susceptibility to enteropathogenic infections of mice calls attention to the need to give due importance to designing diets in mouse studies. Thus, mouse studies investigating the underlying mechanisms of enteropathogen infections should involve a critical assessment of the animal chow compositions across different laboratories. Our observation might have been overlooked in the absence of GF control groups, highlighting the importance of such controls when studying enteropathogenesis mechanisms. At a broader level, our observational study suggests that even given the high potential of dietary modulations via fiber supplementation for the benefit of human health (29), studies should be performed carefully, considering the underlying microbiota composition and acknowledging potential downfalls due to unexpected side effects.

### Ethical statement.

All animal experiments were performed according to the “Règlement Grand-Ducal du 11 janvier 2013 relatif à la protection des animaux utilisés à des fins scientifiques” (30), based on Directive 2010/63/EU on the protection of animals used for scientific purposes (31), and approved by the Animal Experimentation Ethics Committee of the University of Luxembourg and by the Luxembourgish Ministry of Agriculture, Viticulture, and Rural Development (national authorization no. LUPA 2020/27 and LUPA 2019/50). The mice were housed in ISOcages under gnotobiotic conditions in accordance with the recommendations stated by the Federation of European Laboratory Animal Science Associations (FELASA).

### Experimental design and dietary treatment.

For the infection experiments, 6- to 10-week-old, age-matched, male, germfree (GF) BALB/c (*n* = 20, 5 per group) and C57BL/6N (*n* = 31; GF fiber-rich [FR] group, 7 per group; other groups, 8 per group) were housed in ISOcages with up to five animals per cage. Similarly, 6-week-old, female, GF BALB/c mice were housed in ISOcages for time of flight mass cytometry (CyTOF) and short-chain fatty acid (SCFA) measurements (*n* = 12; 6 mice per group for CyTOF, 3 mice per group for SCFA analysis). Light cycles consisted of 12 h of light and sterile water, and diets were provided *ad libitum*. The GF status of the mice was confirmed by aerobic and anaerobic microbial culturing of fecal samples. As per the groupings (Fig. 1A), the relevant mice were gavaged with 0.2 ml of a 14-member synthetic human gut microbiota (14SM) gavage mix on two consecutive days. The gavage mix was prepared as described previously (15). Before and 6 days following the gavage, all mice were maintained on a standard mouse chow which we refer to as a fiber-rich (FR) diet. Afterwards, half of the gavaged and half of the GF mice were switched randomly to a fiber-free (FF) diet, while the rest were maintained on the FR diet. In contrast to the FR diet, the FF diet does not contain dietary fiber from plant sources but instead contains increased glucose levels (4). All mice in the infection groups were maintained for 20 days on their respective diets, while mice from the SCFA and CyTOF measurement groups were maintained for 40 days on their respective diets. Fecal samples were collected once a week during this period. After their 40-day feeding period, mice from the SCFA and CyTOF groups were euthanized by cervical dislocation. Colons were excised and immediately processed for CyTOF measurements (see the details below). Cecal contents from three mice per group were collected and flash frozen before being stored at −80°C for subsequent SCFA measurements (see the details below). After their 20-day feeding period, mice from the infection group were infected with their respective pathogens; BALB/c mice were infected with L. monocytogenes, and the C57BL/6N mice were infected with *S.* Typhimurium. The mice were infected by oral gavage and without any sort of pretreatment, such as antibiotics or fasting. Following the infection, the mice were observed for up to 10 days on their respective diets, and fecal samples were collected daily for all possible mice. Upon reaching the humane endpoint or the end of the 10-day observation time, mice were euthanized by cervical dislocation. Livers and spleens were collected to determine pathogen load and spleen weight. Cecal contents were flash frozen and stored at −80°C for LCN-2 measurements (see the details below). Due to the rapid disease development, it was not possible to reliably obtain fecal material during the course of the *S.* Typhimurium infection because of the severe symptoms; accordingly, we could not compare CFU counts from feces between these groups.

### Animal diets.

The fiber-rich diet was a standard, autoclaved rodent chow (LabDiet, St. Louis, MO, USA; catalog no. 5013). The fiber-free diet was manufactured and irradiated by SAFE Diets (Augy, France) according to the TD.140343 diet formulation, which is a modified version of the Harlan.TD08810 diet (Envigo, Indianapolis, IN, USA) described previously (4). The resulting fiber-free diet lacks all dietary fiber and has an increased glucose content to compensate for the lack of fiber; note that this diet contains crystalline cellulose, although it cannot be degraded by any of the 14SM members.

### Colonization with 14SM.

All 14SM-constituent strains were cultured and intragastrically gavaged as described previously (15).

### Quantification of bacterial relative abundance.

The colonization of individual strains in the 14-member synthetic microbiota was confirmed using phylotype-specific qPCR primers as described previously (15), and the relative abundances of individual microbial strains were computed using the same qPCR protocol (15).

### Pathogen culturing and enumeration.

Both Listeria monocytogenes (Murray et al.) Pirie (ATCC BAA-679) and Salmonella enterica subsp. *enterica* (ex Kauffmann and Edwards) Le Minor and Popoff serovar Typhimurium (strain SL1344, DSM 24522) were grown aerobically in Luria-Bertani (LB) broth. They were grown overnight at 37°C by continuous two-dimensional vortexing in culture tubes using a Skyline RM-2L Intelli mixer at 40 rpm. Cultures were then spun down by centrifugation for 10 min at 5,000 rcf and resuspended in LB broth to reach the appropriate number of CFU for gavage. BALB/c mice were infected with 10^9^ CFU of L. monocytogenes, and C57BL/6 mice were infected with 10^8^ CFU of *S.* Typhimurium. Fecal CFU enumeration was performed as described previously (4), with the modification of the selective media, which differed by strain. Tissue was processed in the same manner, except that the homogenization was performed using a tissue grinder. L. monocytogenes was plated on Oxford agar plates, while *S.* Typhimurium was plated on streptomycin-containing (50 μg/ml) LB agar plates.

### Mouse disease scoring.

A project-specific scoring system based on the FELASA guidelines for reporting clinical signs in laboratory animals (32) was used to determine the mouse disease score. This scoring system is shown in Table 1.

### Intestinal fatty acid analysis.

Thirty to 100 mg of flash-frozen cecal content from uninfected BALB/c mice, which were kept for 40 days on the FR or FF diet, was used for the fatty acid analysis. Per 50 mg of the samples, 500 μl MilliQ water containing 2 mM 2-ethylbutyric acid as an internal standard and 1.4-mm ceramic beads (5 beads per tube) were added. Homogenization was performed for 30 s at 6,000 rpm at 0 to 5°C (Precellys24 homogenizer, catalog no. P000669-PR240-A; Bertin Technologies, Montigny-le-Bretonneux, France), and the resulting homogenate was then centrifuged at 21,000 × *g* for 5 min at 4°C. Further processing of the sample homogenate and measurements were performed as previously described using gas chromatography-mass spectrometry (GC-MS) (33).

### Immune cell profiling of colonic lamina propria.

For the immune cell profiling, uninfected BALB/c mice were kept for 40 days on the FR or FF diet. Colons were excised and placed in Hanks’ balanced salt solution (HBSS) with phenol red and without calcium and magnesium (Lonza, Basel, Switzerland; catalog no. BE10-543F). Lamina propria cells were extracted using a lamina propria dissociation kit (catalog no. 130-097-410; Miltenyi Biotec, Bergisch Gladbach, Germany) according to the manufacturer’s instructions. Cell staining for mass cytometry acquisition was performed according to the method of Guerin et al. (34). Briefly, a total of 3.0 × 10^6^ cells per sample were pelleted into individual 15-ml Falcon tubes and stained with 5 μM cisplatin for 5 min. Cells were washed, and cell surface staining mix containing preconjugated antibodies (Fluidigm, South San Francisco, CA, USA) was added for 30 min at room temperature. Cells were washed twice with flow cytometry (FACS) staining buffer and then fixed using the FOXP3 Fix/Perm kit (eBioscience, San Diego, CA, USA) for 45 min at 4°C, followed by a permeabilization wash. The intracellular staining mix was added to the cells for 30 min at room temperature. Samples were washed twice with FACS buffer, and then pellets were resuspended in cell-ID intercalator-IR in MaxPar fixation solution (Fluidigm, South San Francisco, CA, USA). On the day of acquisition, samples were washed twice with 1× phosphate-buffered saline (PBS) and then twice with deionized water. Samples were resuspended in deionized water at 0.5 × 10^6^ cells/ml with 10% calibration beads (EQ Four Element calibration beads; Fluidigm) and then acquired on the Helios mass cytometer (Fluidigm). Samples were kept at 4°C for a maximum of 5 days before acquisition. Flow cytometry standard (FCS) files were normalized before being imported into FlowJo (BD, Franklin Lakes, NJ, USA). The data were cleaned to remove in the following order beads, doublets, DNA^−^, dead cells, and CD45^−^ cells. CD8^+^ T cells were gated on CD3^+^ CD8^+^ cells. Th1 T cells were gated on CD3^+^ CD4^+^ Tbet^+^ cells. NK cells were gated on CD19^–^ CD3^−^ CD49b^+^ Tbet^+^ cells.

### Lipocalin ELISA.

Samples for the lipocalin enzyme-linked immunosorbent assay (ELISA) were prepared as described previously (4) and measured using the mouse lipocalin-2/NGAL DuoSet ELISA R&D system (Bio-Techne, Minneapolis, MN, USA; catalog no. DY1857) according to the manufacturer’s instructions.

### Detection of bacterial glycan-degrading enzyme activities.

The enzymatic activities of sulfatase, α-fucosidase, β-*N*-acetyl-glucosaminidase, and β-glucosidase were determined using *p*-nitrophenyl glycoside-based enzyme assays from fecal samples as described previously (17).

### Statistical analyses.

Statistical analysis was performed using Prism 8.1.1. (GraphPad Software, Inc., San Diego, CA, USA), except for the bacterial glycan-degrading enzyme activities, for which R Studio (version 4.0.2) with the “kruskal.test” function within the dplyr package (version 1.0.2) and the “compare_means” function in the ggpubr package (version 0.4.0) was used. Statistical significances are represented by asterisks as follows: *, *P < *0.05; **, *P < *0.01; ***, *P < *0.001; and ****, *P* < 0.0001. Unless otherwise specified in the figure legend, for normal distributed values, unpaired two-tailed *t* tests were used, while for nonnormal distributed values, a Mann-Whitney test was used. For multiple comparisons, a two-way analysis of variance (ANOVA) with a Tukey-Kramer *post hoc* test was performed in Prism. The specific test and the number of animals used for each experiment are detailed in the figure legends.

10.1128/mSystems.00717-21.1 FIG S1 CyTOF gating strategy using FlowJo. Data were cleaned to remove, in the following order, beads, doublets, DNA^−^, dead cells, and CD45^−^ cells. Cells were first gated on CD3. CD8^+^ and CD4^+^ T cells were then discriminated using the CD3^+^ gate. Th1 cells were gated from CD4^+^ cells. From the CD3^−^ population, CD19^−^ cells were gated, and from this, NK cells were gated based on expression of Tbet and CD49b. Download FIG S1, EPS file, 2.1 MB. Copyright © 2021 Wolter et al. 2021 Wolter et al. https://creativecommons.org/licenses/by/4.0/ This content is distributed under the terms of the Creative Commons Attribution 4.0 International license.

10.1128/mSystems.00717-21.2 TABLE S1 Comparison of major experimental parameters and readouts between the present study and our previously published work (4). Download Table S1, DOCX file, 0.02 MB. Copyright © 2021 Wolter et al. 2021 Wolter et al. https://creativecommons.org/licenses/by/4.0/ This content is distributed under the terms of the Creative Commons Attribution 4.0 International license.
