# Supplementary material for: Dietary Modulation Alters Susceptibility to Listeria monocytogenes and Salmonella Typhimurium with or without a Gut Microbiota
Source: mSystems. 2021 Nov 2;6(6):e00717-21. doi: 10.1128/mSystems.00717-21 (PMC8562491; doi:10.1128/mSystems.00717-21)
Supplement: TABLE S1 [file msystems.00717-21-s0002.docx]

**Supplementary table 1.** Comparison of major experimental parameters and readouts between the present study and our previously published work (4).

|  | *Listeria monocytogenes* | *Salmonella* Typhimurium | *Citrobacter rodentium* |
| --- | --- | --- | --- |
| **Experimental parameters** | | | |
| Mouse Model | BALB/c | C57BL/6N | Swiss Webster |
| Pathogen infection dose | 10^9^ CFUs | 10^8^ CFUs | 10^9^ CFUs |
| Selective culture medium | Oxford Agar | LB-agar with 50 μg/ml Streptomycin | LB-agar with 50 μg/ml Kanamycin |
| Fiber-rich (FR) diet | Autoclaved rodent chow (LabDiet, 5013) | Autoclaved rodent chow (LabDiet, 5013) | Autoclaved rodent chow (LabDiet, 5010) |
| FR diet manufacturer | LabDiet (St. Louis, Missouri, USA) | LabDiet (St. Louis, Missouri, USA) | LabDiet (St. Louis, Missouri, USA) |
| Fiber-free (FF) diet | TD.140343 (4) | TD.140343 (4) | TD.140343 (4) |
| FF diet manufacturer | SAFE diets (Augy, France) | SAFE diets (Augy, France) | Envigo (Indianapolis, Indiana, USA) |
| Mouse facility location | Luxembourg Institute of Health, Esch-Sur-Alzette, Luxembourg | Luxembourg Institute of Health, Esch-Sur-Alzette, Luxembourg | University of Michigan, Ann Arbor, Michigan, USA |
| Microbiome profiling method | qPCR using strain-specific primers | qPCR using strain-specific primers | 16S rRNA gene Illumina sequencing and qPCR using strain-specific primers. |
| Organic acid and SCFA measurements | GC-MS | GC-MS | HPLC |
| **Readouts** | | | |
| Microbiome composition | Yes | Only before infection | Yes |
| Carbhoydrate growth profiles of 14SM | No | No | Yes |
| Carbohydrate-active enzymes gene expression | No | No | Yes |
| Carbohydrate-active enzyme activity | Yes | Yes | Yes |
| SCFA concentrations | Yes | No | Yes |
| LCN-2 concentration | Yes | Yes | Yes |
| Profiling of immune cell populations | Yes | No | No |
| Colonic mucus layer thickness measurements | No | No | Yes |
| Expression of genes involved in colonic mucus production | No | No | Yes |
| Host cecal transcriptome profile | No | No | Yes |
| Colon length | Not shown  (not significant for all comparisons) | Not shown  (not significant for all comparisons) | Yes |
| Survival | Yes | Yes | Yes |
| Weight | Yes | Yes | Yes |
| Disease score | Yes | Yes | No |
| Fecal pathogen load | Yes | No | Yes |
| Tissue pathogen load | Yes | Yes | No |
| Histopathological scoring | No | No | Yes |
| Localization of pathogen in colon | No | No | Yes |
